# A Suggested Method of Copying Sphygmograms

**Published:** 1898-12

**Authors:** W. Macphun Semple


### A SUGGESTED METHOD OF COPYING SPHYGMOGRAMS.

BY

W. MACPHUN SEMPLE, M.B., C.M. Glasg.

SOME little time ago it occurred to me that by taking sphygmograph tracings on transparent butter paper I should be able to use the resulting tracing as an ordinary photoplate, and thereby be able to make as many accurate copies of any given tracing as might be required and also be able to use the original tracing as a lantern slide.

The method I adopt is very simple. A piece of ordinary transparent tracing paper is cut into strips to suit the particular instrument used, and then smoked over a piece of camphor in the ordinary way. By placing a bottle filler inverted over the camphor and leaving air space at the bottom, a compact column of smoke is obtained, and so a much more evenly smoked paper is produced, with much less camphor, than by burning in the usual way in a teacup or saucer.

When the paper is properly smoked the tracing of the pulse is made at once and varnished. The varnish I use is benzoline and gum dammar, which answers admirably. When the varnish is dry, by holding the tracing to the light, it will be seen that it is quite transparent, with a dark ground, and thus is obtained what is practically a photographic plate, ready for printing. The next step is to get a small printing-frame, and place a piece of clear glass in it. The tracing is then put on the glass, and on that is placed a piece of ordinary sensitised silver paper, or, better still, a piece of ferro-prussiate paper such as engineers use, and having fixed up the back, the printing-frame is exposed for about a quarter of an hour in the sunlight. The last stage is to take the piece of ferro-prussiate paper and wash it well in several changes of cold water, when the tracing will be seen to come out, in dark blue on a white ground. As many of what I may call "photoscripts" of the tracing may be taken as are required, and so the process could be made use of for classes of students, or for exchange with other doctors in connection with a "Sphygmograph Club," or for any of the many purposes for which tracings may be required. I consider the "photo-script" is better than the original, as it gives the tracing in dark colour on a white ground, and so the curves and differences are seen to greater advantage than when in white on a black ground as in the original. There is no technical skill whatever required, and the only thing I should wish specially to be noted is that the paper must be more thickly smoked than usual, and when smoking, the paper must be kept sufficiently far away from the flame not to harden in the smoke and so prevent the needle of the sphygmograph from making a well-defined track through it.

The copies can be more quickly made by gaslight; only in that case bromide paper must be used, which gives a blackish brown tracing on a white ground. The exposure in that case would be ten seconds for each copy.

### Progress of the Medical Sciences.

